# Perspectives on and Experiences With Remote Monitoring and Patient-Initiated Care Among Norwegian Patients With Axial Spondyloarthritis: Qualitative Study

**DOI:** 10.2196/63569

**Published:** 2025-03-28

**Authors:** Christine Hillestad Hestevik, Cecilie Varsi, Nina Østerås, Anne Therese Tveter, Jon Skandsen, Hedda Eik

**Affiliations:** 1 Health Services Research and Innovation Unit Diakonhjemmet Hospital Oslo Norway; 2 Centre for Treatment of Rheumatic and Musculoskeletal Diseases (REMEDY) Diakonhjemmet Hospital Oslo Norway; 3 Division of Health Services Norwegian Institute of Public Health Oslo Norway; 4 Faculty of Health and Social Sciences University of South-Eastern Norway Drammen Norway; 5 Department of Public Health and Interdisciplinary Health Sciences University of Oslo Oslo Norway; 6 Department of Rehabilitation Science and Health Technology Oslo Metropolitan University Oslo Norway; 7 Patient Council Centre for Treatment of Rheumatic and Musculoskeletal Diseases (REMEDY) Diakonhjemmet Hospital Oslo Norway

**Keywords:** remote monitoring, patient-initiated care, patient-reported outcome measures, chronic disease, rheumatology, axial spondyloarthritis, joint disease, spine, medication, therapy, rheumatic, patient care, randomized controlled trial, interventions, decision-making

## Abstract

**Background:**

Axial spondyloarthritis (axSpA) is a chronic inflammatory joint disease affecting the spine and sacroiliac joints, requiring frequent, lifelong monitoring and treatment. This involves regular symptom monitoring, assessing medication tolerance and side effects, and prompt therapy adjustments. Typically, patients with axSpA attend prescheduled hospital visits, but once stable disease has been attained, these seldom align with periods of high disease activity. Remote monitoring and patient-initiated care offer flexible, need-based, follow-up options. However, knowledge about how patients with axSpA perceive and experience these approaches is limited. To effectively implement these strategies in clinical practice, understanding patient perspectives is crucial.

**Objective:**

This study aims to explore how patients with axSpA perceive and experience remote monitoring and patient-initiated care.

**Methods:**

Our qualitative study was embedded in a randomized controlled trial. Participants were allocated to either usual care, remote monitoring, or patient-initiated care. The 2 intervention groups had no prescheduled visits and used a remote monitoring app, but only the remote monitoring group received monitoring by health care professionals. Semistructured interviews were conducted with 18 participants from the intervention groups to explore their experiences. The interviews were audio recorded, transcribed, anonymized, and analyzed using thematic analysis. Participants provided informed consent.

**Results:**

Eighteen patients (11 men and 7 women, aged 26-65 years) participated, 10 from the remote monitoring group and 8 from the patient-initiated care group. Transcripts were analyzed into four key themes: (1) “I don’t need to go to the hospital just to report I’m doing well.” When patients felt well, they perceived in-person consultations as less important. They acknowledged health care resource challenges and were willing to adapt but expressed concerns about rapid technological advancement, fearing it could exclude vulnerable groups. They emphasized the need for shared decision-making in determining follow-up strategies; (2) “It feels safer to meet healthcare personnel in person” highlighted participants’ preference for in-person interactions as a safety net for detecting changes or signs of disease. They felt more secure when communicating face-to-face with health care professionals; (3) Remote monitoring can promote a sense of freedom and self-efficacy. The app provided autonomy, enabling patients to monitor their health without disrupting daily routines and promoting their health competency; and (4) Practical challenges and limitations of technology affect sense of security. Concerns about app deactivation, digital privacy, and lack of personalized settings negatively affected confidence in technology and sense of security.

**Conclusions:**

Remote monitoring and patient-initiated care can adequately meet the needs of patients with axSpA with low disease activity, reducing unnecessary visits and enhancing self-efficacy. However, these approaches should not be one-size-fits-all. Care must adapt to evolving disease activity, circumstances, and preferences. Human interaction and support remain crucial, and future technological developments must address practical challenges to ensure user-friendly and reliable interfaces.

## Introduction

Axial spondyloarthritis (axSpA) is a chronic inflammatory joint disease that predominantly affects the spine and sacroiliac joints [1]. The disease manifests symptoms such as chronic back pain and stiffness over weeks or months, along with early-morning stiffness and pain that diminishes throughout the day with physical activity. Fatigue and tiredness are also common symptoms. The disease typically begins in early adulthood, often in the teens and those in their twenties, and can potentially result in irreversible joint damage and functional disability [2]. While there is currently no cure for axSpA, significant advancements in management and therapeutic strategies over the last few decades have led to substantial improvements in the prognosis for most patients with inflammatory joint diseases, including those with axSpA [3,4].

The primary goal of treatment is achieving inactive disease (remission) or low disease activity [2]. However, disease activity tends to fluctuate over time, and many patients experience episodes of significant worsening of symptoms, known as disease flares. A flare is generally considered an increase in disease activity that may necessitate a change or intensification of therapy [5]. Consequently, the management of axSpA requires frequent and lifelong monitoring and treatment [6].

Some patients with axSpA use disease-modifying antirheumatic drugs, such as tumor necrosis factor inhibitors, and receive long-term follow-up in specialist health care with assessments of treatment efficacy [7,8]. This comprehensive approach involves regular monitoring of clinical symptoms, assessing the tolerance and side effects of medications, and making prompt therapy adjustments in a treat-to-target strategy [6]. The evolving landscape of therapeutic strategies and the emphasis on early intervention aim to enhance the quality of life for individuals with axSpA, underscoring the importance of ongoing vigilance and personalized care [4,6].

In Norway, patients with inflammatory joint diseases such as axSpA involve scheduled hospital visits according to a standardized time schedule. Patients with axSpA are usually offered regular, prescheduled, face-to-face visits with a rheumatologist or rheumatology nurse at the hospital’s outpatient clinic and have the possibility to request extra visits if they experience increasing symptoms.

Several studies have indicated that scheduled visits often do not align with periods of high disease activity [9-11]. A study conducted in the Netherlands revealed that 34% of spondyloarthritis outpatient visits were considered unnecessary by rheumatologists and that 51% of all the visits did not lead to any clinical action [11]. Frequent, and often untimely, clinical visits impose both a financial burden on society and demand significant time from the patients [11,12]. Therefore, a more personalized and adaptive approach to the management of inflammatory diseases, such as axSpA, is imperative.

Remote monitoring and patient-initiated care can be part of the solution to these challenges, as they offer a more flexible and need-based follow-up for this patient group. Remote patient monitoring uses technology that enables monitoring of patients outside traditional clinical settings, such as at home or in remote areas. It involves transmitting health data directly to care providers through automated electronic means or web- or smartphone-based data entry, enabling prompt intervention if symptoms worsen [13,14]. Patient-initiated care involves giving patients and their carers flexibility to arrange their follow-up appointments as and when they need them based on symptoms and individual circumstances [15]. These approaches contrast with the calendar-based regular hospital appointments, allowing for tailored treatment based on individual needs and disease activity. This can yield beneficial effects for both individuals and society by reducing the burden on individual patients and minimizing the number of unnecessary visits [16,17]. In Norway, remote delivery of care is a strategic priority to achieve more sustainable health care services [18,19].

Despite the potential benefits, there is some reluctance among patients to use and adhere to remote monitoring. Two systematic reviews of qualitative studies indicate that remote monitoring has the potential to improve self-efficacy, disease-specific knowledge, and shared decision-making. However, these advantages are counterbalanced by concerns about trust in technology and the potential loss of interpersonal contact [20,21]. Patients expressed that personal contact was instrumental in establishing trust and fostering better communication [21]. Results from studies on patient-initiated care have also highlighted challenges. Patients have concerns about the responsibility of self-monitoring and initiating their own care and concerns related to underreporting and under-diagnosis [12,22].

However, there is a notable gap in knowledge regarding how patients with axSpA perceive and experience remote monitoring and patient-initiated care. To successfully implement these new follow-up strategies for patients with axSpA and other chronic conditions in clinical practice, a comprehensive understanding of their perceptions and experiences is crucial. We aim to provide insights to inform the effective implementation of remote follow-up strategies in rheumatic patient care by exploring: How do patients with axSpA perceive and experience remote monitoring and patient-initiated care?

## Methods

### Study Design

Our study is related to an ongoing randomized controlled trial (RCT) that is investigating remote monitoring and patient-initiated care for patients with axSpA. This is a qualitative study applying data from individual semistructured interviews with some of the participants from the RCT to explore their perspectives on and experiences with remote follow-up and patient-initiated care. We adhered to the Consolidated Criteria for Reporting Qualitative Research (COREQ; Multimedia Appendix 1) [23].

### Setting

The study setting was the ReMonit trial [24], a single-center RCT conducted at a rheumatology unit within a Norwegian hospital between September 2021 and December 2023. The RCT aimed to test whether remote monitoring and patient-initiated care were noninferior to usual care. The digital platform Dignio for health care personnel and the MyDignio patient app software enabled remote monitoring. Of 346 screened patients, 243 were enrolled between September 2021 and June 2022 and randomly allocated 1:1:1 to either usual care (n=82), remote monitoring (n=80), or patient-initiated care (n=81).

Usual care entails adherence to the current conventional follow-up regimen, involving patient-reported outcome measures (PROMs), blood tests, and hospital visits every 6 months with an experienced rheumatology nurse or rheumatologist.

The remote monitoring study arm incorporated the use of remote monitoring and care through the MyDignio platform and software, facilitating the remote collection of PROMs, patient monitoring, triaging via the clinicians’ dashboard, and asynchronous chat. The MyDignio platform facilitated the digital transfer of health data between patients and health care personnel. Additionally, participants received SMS reminders for “tasks” once a month such as self-reporting PROMs or uploading blood test results. A subgroup used a C-reactive protein (CRP) instrument for home-based measurements.

In the patient-initiated care arm, participants did not undergo remote monitoring and had no prescheduled hospital visits. Instead, they were instructed to contact the hospital if they experienced significant worsening of symptoms and deemed a consultation with a health professional necessary. These participants had the MyDignio app and received SMS reminders for self-reporting PROMs every third month, but the data were not monitored. However, they could communicate with the hospital through the chat function in the app.

Participants in all 3 arms were instructed to take regular blood tests due to the medications they were prescribed and had direct telephone access to a specialist nurse at the hospital during the study period.

### Recruitment of Participants

Eligibility criteria for the main study (ie, the RCT) included a stable medical treatment history with tumor necrosis factor inhibitors for the last 6 months and low disease activity (defined as Axial Spondyloarthritis Disease Activity Score <2.1) at the time of inclusion in the trial. Additionally, participants were required to speak Norwegian and be able to provide informed consent. Exclusions were made for patients with major comorbidities, as well as pregnant and breastfeeding women. This information is described in more detail in the study protocol for the RCT [24].

The participants in this qualitative study comprised adults diagnosed with axSpA who were randomized into one of the 2 intervention groups (remote monitoring or patient-initiated care) in the ReMonit trial. All the participants in the RCT consented to share their experiences with the follow-up through interviews. Purposive sampling was used to ensure a diverse representation of participants in our qualitative study, considering factors such as sex, age, amount of contact with study personnel, and allocated intervention group. A purposive sample was contacted by phone by the first author and invited to participate.

### Data Collection

Demographic patient data were collected as part of the ReMonit trial through anamnesis and physical examinations by a rheumatologist, self-reports by patients in digital questionnaires, and laboratory assessments of blood samples.

We developed an interview guide based on previous research on patient experiences with remote follow-up strategies, clinical experience, and input from a patient research partner and qualitative research experts. The interview questions covered topics related to perspectives on and experiences with their follow-up, the use of digital tools for symptom monitoring, and how this affected therapeutic interactions and communication (Multimedia Appendix 2). We endeavored to design open-ended questions that encouraged participants to provide rich, detailed descriptions of their experiences. We began the interviews and continuously evaluated the interview guide, discussing whether adjustments were necessary as we progressed. No major changes were made to the questions. The interviews were conducted between June 2023 and September 2023.

The first author, who was employed as a postdoctoral researcher at the hospital, conducted the data collection through semistructured interviews. Interviews were continued until the sample provided sufficient information power, indicating that the data held sufficient information to address the study aim, considering both the information held by the sample and the contributions of new knowledge derived from the analysis [25]. After 18 interviews, the first author found that the interviews were rich and varied in descriptions, providing comprehensive insights into the participants’ experiences and perspectives. We also observed that the final 3-4 interviews yielded minimal new information, confirming that the scope and detail of the analysis were sufficiently exhaustive to support robust conclusions.

Interviews were conducted 1-3 months after the participants’ end-of-study hospital visit. The interviews took place either at the hospital (n=2) or on a secure videoconferencing platform (n=16), depending on the patient’s preferences. The interviews lasted between 17 minutes and 1 hour. All interviews were audio recorded and transcribed verbatim and anonymized.

### Data Analysis

The data were subjected to thematic analysis in an iterative and inductive process, drawing guidance from Braun and Clarke approach [26]. The analysis team constituted the first author (CHH), the second author (CV), and the last author (HE). Initially, the team engaged in an in-depth reading of the data to familiarize themselves with the data and collectively discussed the overall impressions related to the research question.

Subsequently, CHH manually coded each interview to identify and label key features of the data that were relevant to the research question. Throughout the analysis, the team convened multiple times in an iterative process to discuss general impressions and emerging themes that resonated with the overarching study aim.

This ongoing process, involving multiple rounds of analysis, enabled us to refine the initial codes and adjust the thematic structure by merging or splitting themes based on their relevance and clarity. The process further led to the identification of four key themes that encapsulated significant patterns across the dataset.

The final phase of our analysis involved selecting expressive quotes from the transcripts to exemplify our analytical points. To craft the analytic narrative, all authors collaborated, ensuring a comprehensive and coherent representation of our findings. The narrative was further enriched by inputs from a patient research partner who provided valuable comments on various drafts. We used the software NVivo (Version 14; Lumivero) to organize and analyze the data.

### Ethics Approval

The Regional Committees for Medical and Health Research Ethics in Norway granted approval for the study (229187). Participants provided informed consent to participate in the main RCT and in the qualitative interview upon completion of the trial. Participants were not provided with any compensation for participating in this study. Audio recordings and transcribed interviews were securely stored on the services for sensitive data facilities, owned by the University of Oslo and operated and developed by the Services for Sensitive Data service group at the University of Oslo, IT-Department [27], in compliance with the research rules and guidelines at the hospital.

## Results

### Description of the Participants

A total of 18 patients (11 men and 7 women, aged 26-65 years) were invited and agreed to participate in the interview study, with 10 in the remote monitoring group and 8 in the patient-initiated care group (Table 1).

**Table 1:** Characteristics of the participants (N=18).

Characteristics			Remote monitoring		Patient-initiated care
Participants, n (%)			10 (56)		8 (44)
Sex (female), n (%)			3 (30)		4 (50)
Age (years), mean (SD)			46.9 (14.7)		50.9 (7.6)
**Number of contacts with health care professionals, n (%)**					
	0	5 (50)		5 (63)	
	1–2	3 (30)^a^		3 (38)^b^	
	More than 2	2 (20)^c^		0 (0)	

^a^Three patients with 5 telephone consultations.

^b^Three patients with 3 telephone consultations and 1 hospital admission.

^c^Two patients with 7 telephone and 2 in-person consultations.

Many of the patients reported that they had previously consulted various health care professionals, before getting the necessary help to manage their disease. Receiving the appropriate diagnosis and treatment with biological medication proved to be life-changing alleviating most of their symptoms. Consequently, for most of the participants, the disease no longer had a significant impact on their daily activities, except for the necessity of having to take medication. However, some of the patients experienced more challenges such as sleep deprivation and fatigue, affecting their daily lives and, in certain instances, their ability to work.

Although 2 interventions were involved, both groups used the remote monitoring app including the chat function. This led to similar experiences between the 2 groups. While only the remote monitoring group used the actual monitoring feature, the patient-initiated care group still had thoughts and perspectives related to their use of the app and remote care. Patients in the remote monitoring group were informed that their PROM data would be routinely monitored, whereas the patient-initiated care group was told that their PROM data would not be monitored but would be included in data collection for research purposes. Despite being informed of this upon entering the study, some members of the patient-initiated care group seemed unaware that they were not actively monitored via the app.

Our analysis of the patient interviews resulted in four main themes: (1) “I don’t need to go to the hospital just to report I’m doing well,” (2) “It feels safer to meet health care personnel in person,” (3) remote monitoring can promote the sense of freedom and self-efficacy, and (4) practical challenges and limitations of technology affect sense of security.

### “I Don’t Need to go to the Hospital Just to Report I’m Doing Well”

Overall, the participants in both groups were aware of the challenges posed by limited resources in the health care system and the need for prioritization to conserve staff resources. They were willing to contribute to alleviating the situation as the following quote suggests.

I did tell her [the doctor], that, you don’t need to spend too much energy on me. I can contact her if there’s any issue. There are probably many patients who have a greater need to talk to the doctor than me. ...I know, the situation in Norway today is that there’s pressure on the health care system, and so I’m trying to be flexible and say, well, you don’t need to use a lot of resources on me.

Woman, 49 years, patient-initiated care

Most of them strongly believed in the use of technology to manage patients like themselves. When patients felt well and perceived their illness as having little impact on their daily lives, they viewed regular in-person consultations as less important. During these times, they felt more confident in managing their condition on their own and did not see the need to go to the hospital just to report “I’m doing well.” However, patients in the remote monitoring group recognized the limitations of remote monitoring, especially during disease flare-ups. As 1 participant put it.

So like as soon as my score on the app worsens [health deteriorates], I’m all for a person, but as long as I’m doing well, I don’t feel the need to come here and say that I’m doing well...I think the most important thing revolves around the app phobia and the societal issue that one needs to be careful not to automate away from all human contact. That it’s a good tool for now if it’s okay for the patient, as with all diseases, if you’re doing well, you don’t need to talk to the doctor. If you can’t automate, you can at least rationalize a lot of it, but doctors are good to have when you need someone to talk to. Both when you’re sick and maybe struggling with heavy thoughts.

Man, 55 years, remote monitoring

If their illness progressed or if they developed additional health issues the participants in both groups expressed a greater need for the doctor’s presence to discuss their situations more freely and to receive emotional support. On the other hand, some deliberated on whether the social and relational aspects of health care should be considered part of the health care system’s responsibilities in the case of limited resources as this quote suggests.

If we take a broader perspective, I do think that some people might need to meet others physically to fill their days with activities and social interactions. However, at the same time, that’s not what hospitals should be used for. I’m not in favor of the trend that we might soon not meet in person at all. Still, I do think it’s much more efficient.

Woman, 45 years, patient-initiated care

Participants from both groups had concerns about the rapid advancement of technology in health care and voiced apprehensions about the technology’s potential to exclude vulnerable patient groups. They emphasized the necessity for shared decision-making in determining the appropriateness of remote monitoring and patient-initiated care, and that such decisions should be negotiated on a case-by-case basis. They also stressed the importance of tailoring the follow-up based on individual preferences, needs, changes in disease activity, and evolving life circumstances as this quote implies.

Everyone with these diseases is different, so I think that perhaps what one needs varies, and in twenty years maybe I will say something completely different than what I’m saying now, because this has worked very well for me. But I do think that perhaps it won’t work for everyone. We are all different and need different things.

Man, 52 years, patient-initiated care

### “It Feels Safer to Meet Healthcare Personnel in Person”

A crucial factor for feeling secure for the patients in both intervention groups was easy access to health care professionals. They generally felt confident in taking more responsibility for their care as long as health care personnel were responsive and available when needed. A participant in the remote monitoring group highlighted that it was reassuring to place the responsibility on the doctor to monitor the PROMs and reach out in case of missed notifications, tests, or reported changes in symptoms as this quote suggests.

I mean, it’s very simple, isn’t it? It’s very straightforward. It’s answering some questions, and then, at least in my mind, you’re placing the responsibility on the doctor by just responding to some questions in a way. At the same time, you’re keeping track of your own condition too.

Woman, 40 years, remote monitoring

Both groups also found it easy and convenient to communicate with health care personnel through the chat function in the app. However, the participants generally placed more trust in face-to-face interactions with health care personnel compared to chats, telephone, or video. With remote consultations, they felt that some of the human aspects were being lost as this participant described.

Patient: I was called in to see the doctor at the end of the study. As I told him, I think it’s okay to have an in-person appointment once a year. Just having it digitally feels a bit odd to me. So, I think it’s perfectly fine to have a follow-up session with the doctor/nurse once a year.

Interviewer: Why do you think it’s important?

Patient: Well, it has something to do with me. That personal contact can never be replaced by the digital. I know it has to do with me because I like to talk to people in person. Having a FaceTime [digital consultation] is okay, but meeting people in person is incredibly pleasant, it creates a different atmosphere. It feels maybe a bit safer when someone sees you and can sense your presence. Especially with doctors, perhaps; you can’t achieve that over the phone. You can talk privately with people, but it’s never as personal as an in-person meeting“.

Woman, 65 years, remote monitoring

With remote consultations more responsibility was placed on their own shoulders, and they worried about their own ability to identify and convey important symptoms or health issues digitally. Several examples of situations where health issues had been misinterpreted or not identified in digital communication were cited.

Although the participants in the remote monitoring group had accepted and felt comfortable with remote follow-up, it was evident that most of them firmly believed it could not entirely replace in-person consultations. The majority expressed a preference for continuing to have an in-person appointment at the hospital approximately every, or every other, year.

Many of the patients in the patient-initiated care group also recognized the need for regular in-hospital consultations. However, some patients felt comfortable without any prescheduled appointments and did not see the need for regular visits, as long as they had easy access to the hospital in case the disease worsened.

For many of the participants, regular in-person follow-ups were considered a safety net for detecting changes or signs of a disease. Several participants described how face-to-face discussions during appointments allowed for a more comprehensive assessment of their situation and sometimes led to the discovery of overseen health issues. They emphasized the importance of doctors interpreting body language and noticing subtle signs conveyed by patients. Moreover, many underscored the significance of physical examinations of the joints in the context of dealing with axSpA, as this participant describes.

It’s something about seeing the whole me. I feel that when you sit face-to-face and talk with them, and if you have something like ‘can you check my knees now’ or ‘can you check my fingers,’ you feel that you have a little more contact. I feel a bit safer, in a way. I feel that it’s nice to be able to talk to someone also...if it’s just like that [digitally], then I feel that I lose a bit of connection with the hospital. I feel that they don’t take care of me in the same way, if you understand.

Woman, 61 years, patient-initiated care

The absence of physical touch in the remote consultations made this patient feel as though the doctor had not thoroughly examined all aspects of her disease.

Many participants in both groups also expressed concerns that remote care might lead to a perceived sense of too much distance from health care personnel and the potential elimination of human contact in health care interactions. One participant noted that sharing personal information with strangers through the chat felt uncomfortable, prompting a desire for introductions or presentations of the responding health care personnel and suggested improvements to make the experience somehow more personal as this quote implies.

Interviewer: How would you say this type of follow-up has affected your relationship with health care providers?

Participant: I have perhaps had a somewhat distant relationship with whoever it is I’m talking to, and I don’t think I have seen the person I’ve chatted with, for example, I haven’t seen her face. So, that’s something I’ve thought could be nice, that you almost got a presentation of the people I’m going to chat with, so it feels a bit more personal. And that there could be a profile picture or something like that.... There were basically two people who had access to the app, and I sometimes wrote personal things there, so I think it’s nice to have a personal relationship with them too since I’m sharing quite a bit of information.

Woman, 32 years, remote monitoring

Participants in both groups also emphasized the need for a clear follow-up plan, providing clarity on their responsibilities in managing their own care during the follow-up process.

### Remote Monitoring Can Promote the Sense of Freedom and Self-Efficacy

Most participants in the remote monitoring group appreciated the convenience and time-saving aspects of the follow-up, allowing them to report from home instead of traveling across the city to visit the hospital. They appreciated not having to take time off from work for routine follow-ups, as described by this participant.

The biggest change is that I no longer have to go to the hospital physically, and I also don’t have to go during working hours because consultations are usually scheduled during work hours. This way, I feel more comfortable with my employer because I can respond to this in my free time. I don’t need to spend time getting to the hospital, waiting, having an appointment, and then returning.

Woman, 40 years, remote monitoring

With remote follow-up, the patients appreciated that their condition became less visible to their employers and did not negatively affect their jobs to the same degree as with traditional follow-up. The patient-initiated care group reported similar experiences and found it convenient to contact the hospital themselves when needed to avoid unnecessary hospital visits and time off work.

They also highlighted that when not feeling well, traveling back and forth to the hospital could be a struggle and that many issues could easily be resolved digitally. The reduced time spent on hospital visits granted them more freedom and the disease occupied less space in their lives. This, in turn, allowed them to shift their focus away from the disease and lead more “normal” lives, as these quotes indicate.

By getting this remote home monitoring, I feel freer in a way, even though the follow-up I’ve had so far has been quite minimal because everything has been fine, I can just go on and deal less with the disease....

Woman, 32 years, remote monitoring

Interviewer: How do you feel about being followed up like this?

Patient: It has been incredibly nice because I feel much less pathologized. ...So, I haven’t really been to consultations except for that final consultation, so I hadn’t been there for a year and a half, and it was a very nice feeling.

Woman, 40 years, remote monitoring

On the other hand, self-monitoring could also result in more bodily awareness and interest in getting to know the disease. Some individuals from both study groups perceived the app as a valuable tool for becoming more self-efficient. They appreciated having more control of their own follow-up and saw the app as a potential platform for gaining deeper insights into their disease. Accessing test results and tracking disease progression enabled them to monitor changes over time, empowering them to better understand decisions made by doctors, including medication adjustments as this participant suggests.

I find it very enjoyable to take control of it myself. It’s not like when I’ve had blood tests before, I haven’t cared about the results. I’ve been following closely, and I’ve been doing it for years, and my CRP levels are completely normal, my liver values are fine, and my iron levels are okay. So, I find it quite fun to be a bit more active in it. Not just sending the blood tests to the hospital and then coming in, and the doctor saying everything is fine, but I don’t get to see the results in a way. I find it very enjoyable, at least for myself. ...Yes, I think it’s good, and I can see that if I were worse, for example, or had more fluctuations, I would have been even more concerned about having that control. This way, I also understand what’s happening and why decisions are made to change medication or so on. I find it interesting to be a bit active in it because, after all, it’s a part of my life.

Woman, 32 years, remote monitoring

This participant, equipped with a CRP instrument at home, found joy in gaining a deeper understanding of the disease and actively participating in her own follow-up. To get better oversight and control of the disease, another participant suggested the inclusion of a diary function in the app to monitor potential disease triggers such as activities or foods that resulted in worsening symptoms.

### Practical Challenges and Limitations of Technology Affect Sense of Security

Most participants in both groups believed that the app comprised the necessary functions and appreciated that it was easy to use. They were also satisfied with the regular task and valued the simplicity of answering only two short questions in the app when feeling well, and that follow-up questions were prompted if they reported problems. It was important to them not to be overwhelmed with questions and notifications as this participant suggests.

I answered some questions, and then it was done, because everything was good. ...I didn’t have to fill out a bunch of stuff and say, for the tenth time in these six or seven years I’ve had the illness, no, I don’t have any symptoms, no, I’m not stiff in the morning, I don’t have..., because it’s mostly the same every time I have to answer these questions. I thought it was good.

Man, 26 years, remote monitoring

Some of the participants in both groups felt uncertain about sharing private information digitally due to a lack of trust in the safety of digital solutions. Participants in both groups also encountered technical challenges with the app which negatively affected their care experiences and sense of security. A major concern was the need to repeatedly log into the app, which was deactivated after a certain period. Due to security protocols, they had to log-in via the national secure digital identification system, and for some, this process felt time-consuming, and overly complicated, creating a barrier to adherence. Some participants expressed uncertainty about receiving all messages from the hospital and worried about being disconnected from the “system” due to the app frequently deactivating as this quote suggests.

When you don’t hear anything for a while, you wonder if it’s because they haven’t said anything, or if something has gone missing along the way. ...because there’s also a thing with an app on the phone that monitors active and inactive apps, and if you’re not active for a while, it puts it in sleep mode, and deep sleep, and, I don’t know what it’s all called. But then you start to wonder if this app goes into deep sleep, and they try to communicate with me. Will they get through, or will it get lost along the way? Should I, even if I have nothing to say, go into the app so that it doesn’t go into deep sleep and such things?

Man, 44 years, remote monitoring

The absence of updates in the app could lead to a feeling of being overlooked by the hospital.

Participants in both groups also emphasized the significance of receiving notifications through multiple platforms, including SMS or email, in addition to the app, to prevent missing communication from the hospital. Moreover, many participants expressed the view that the follow-up would be enhanced if it were possible to customize the app settings. They suggested individualizing the frequency and timing of notifications throughout the day and adjusting the response deadline for notifications. Participants also frequently mentioned feeling pressured by the short response deadline for inquiries (2 hours) and expressed a desire for the option to receive notifications a day or two in advance to better prepare.

## Discussion

### Principal Findings

The aim of this study was to explore how patients with axSpA perceive and experience 2 new follow-up strategies: remote monitoring and patient-initiated care.

Participants generally found both follow-up strategies satisfactory if their condition was well controlled. They were aware of the challenges with limited and overstretched resources in the health care system and were willing to contribute by taking up less of health care personnel’s valuable time. These follow-up strategies also had advantages for themselves. It gave them more free time, eliminated the need to take time off work, and allowed them to shift their focus away from the disease. Some also appreciated the opportunity to become more self-efficient and health competent. However, they were skeptical about technology’s rapid advancement and preferred face-to-face discussions for emotional support and safety. Barriers to remote monitoring also included a lack of trust in technology and technological problems.

Patients in both groups were concerned about the accuracy of digitally conveyed information, fearing potential misinterpretations by health care professionals, and felt safer with in-person consultations, which allowed for a more comprehensive assessment. Remote monitoring and patient-initiated care place greater responsibility on patients to recognize symptoms and communicate accurately. This can be challenging for patients with axSpA, as the condition manifests in various ways, and symptom perception varies. Additionally, patients with axSpA may experience side effects and comorbidities like inflammatory bowel disease, psoriasis, or reactive arthritis [2], which may complicate symptom identification. A systematic review of patient-initiated care for chronic conditions found it most suitable when patients can easily identify clinical problems requiring advice [28]. This, along with our findings, underscores the importance of ensuring that patients feel competent in recognizing and reporting relevant symptoms, especially in diseases with diffuse and varying symptoms.

On the other hand, our results suggest that remote monitoring has the potential to enhance the patients’ knowledge and understanding of their disease. Specifically, participants in the remote monitoring group appreciated gaining a better overview of test results and potential disease triggers in the app, enabling them to become more active self-managers. This finding aligns with other studies that have demonstrated how remote monitoring can support and promote self-efficacy and shared decision-making [21,29]. Discussing monitoring data with health care professionals can further empower patients and make them feel like equal partners in their care [21]. This aspect seems crucial, as an umbrella review by Taylor et al [20] revealed that patients with chronic diseases may experience anxiety if provided excessive disease-related information without professional interpretation. Our results, along with these previous findings, indicate that a remote collection of PROMs can promote health literacy if patients are sufficiently involved in the follow-up.

Participants in both study groups also emphasized the importance of receiving emotional support from health care professionals and expressed concerns that remote follow-up might dehumanize care. This underscores the significance of addressing psychosocial aspects of care when implementing new follow-up strategies, which is also supported by other studies. The study by Ekstedt et al [30] explored experiences with telemonitoring of chronic conditions. They found that conversations extending beyond health-related issues helped build trust and security, reducing the risk of misunderstandings and errors. A study of patient-initiated care for patients with chronic or recurrent conditions, also found that a good patient-provider relationship was crucial for feeling confident in self-managing their disease [28].

Participants in both study groups expressed a preference for regular in-person consultations if their condition worsened. However, other studies have shown that remote monitoring can also benefit patients with high disease activity. Kempin et al [31] found that older patients with axSpA with high disease activity had better adherence to a health app and were more open to new tools. Regularly reporting PROMs via an app can prompt medical appointments if the disease worsens. Similarly, Jones et al [32] found that patients with axSpA were more likely to adhere to daily self-tracking when feeling worse, although some discontinued use when symptoms became unmanageable, either due to disease impact or increased focus on negative aspects. Kempin et al [31] reported an adherence rate of less than 30% after 6 months, emphasizing the challenge of sustaining long-term engagement with eHealth apps. This underlines the importance of addressing the barriers to adherence when considering broader implementation of remote monitoring or patient-initiated care. Ensuring adequate support, including backup and escape options, is crucial to prevent loss of follow-up and ensure that patients receive the necessary care. Clearly, patients’ needs evolve with disease activity and life circumstances, requiring adaptive follow-ups. Our results indicate that easy access to in-person consultations when needed can promote reassurance and security during remote follow-up.

Our participants also expressed concerns that some patient groups may be excluded from care due to a lack of digital competence or willingness to use technology. The use of technology may enhance access for some groups while impeding it for others. A study by Müskens et al [33] reported that a Dutch eHealth platform for patients with rheumatoid arthritis was selectively used, primarily by younger and highly educated patients. This shows that digital care solutions potentially can exacerbate existing social health inequalities. Our findings, along with those from previous studies, underscore the importance of carefully evaluating the impact of remote care not only for those who accept and engage in it but also for those who withdraw or are excluded from it. Our results also indicate that increasing patients’ health literacy may make them more confident in self-monitoring their symptoms and may promote engagement in their care.

Our patients appreciated that the app was simple and easy to use, but technical problems and lack of personal customization of app settings were concerns that negatively affected their care experience and adherence to the follow-up. Studies show that design features, usability, and device maintenance impact adherence to health technology devices [34,35]. Poor interfaces and the difficulty and effort of data entry may negatively affect adherence. Similar to our findings, Jones et al [32] found that patients with axSpA valued a digital tool that was customized to individual needs and offered multiple tracking options. Involving patients in the development of health technologies can help identify unmet needs, improve usability, increase adoption and engagement, enhance health outcomes, foster advocacy and trust, and promote health equity and access [36]. Allowing patients to customize their digital follow-up and app features can further increase patient engagement and the effectiveness of remote care [37]. Therefore, patients should be involved in the development and adaptation phases to create apps that are easy to use, with necessary functions and user-friendly interfaces.

### Strengths and Limitations

This study used specific patient selection criteria and reached out to patients receiving care for axSpA within an RCT at a single hospital in a Norwegian urban setting. Additionally, only patients with low disease activity were included in the trial. We recognize that patient populations with more severe diseases or those living in different regions and care settings may encounter different experiences that are not captured in this study. For instance, potential benefits regarding increased care accessibility and time savings may be more significant in rural settings. Furthermore, patients with a high level of health literacy may have been more likely to participate in the RCT and those with strong opinions on remote care may have been more inclined to take part in the interviews. Therefore, the representativeness of the findings and conclusions should be interpreted with caution and warrants further investigation. Additional perspectives from other patient groups and stakeholders, such as health care personnel, could provide valuable insights into this topic. The authors of this study are currently conducting interviews with health care personnel involved in the ReMonit study regarding their perceptions of the 2 follow-up strategies. The results from these interviews will be presented in a separate publication.

The inclusion of regular symptom reporting through the app in both interventions made it difficult to clearly differentiate between the experiences of the 2 intervention groups. The patient-initiated care group shared many thoughts and perspectives about their use of the app and remote care that were similar to those of the remote monitoring group. Additionally, some patients were unaware that they were not being actively monitored, which may have influenced their perceptions. Future studies should consider this overlap to better distinguish the experiences and outcomes between these two follow-up approaches.

A strength of the study is the participants’ 18-month experience with the follow-up strategies and app usage, offering data based on prolonged use. We believe that this qualitative approach has generated valuable findings that can be of interest also to patients with other chronic diseases using various remote care apps and follow-up strategies. Furthermore, we think our findings can contribute to the evolution and improvement of practices related to remote monitoring and patient-initiated care strategies for patients with chronic conditions.

### Reflexivity

Researchers have a subjective influence on the research process and its findings and reflexivity acknowledges the role of the researcher as a participant in the process of knowledge construction [38]. CHH, CV, HE, and ATT had no role in the treatment of the participants and had no prior relationship with them before this study. NØ was involved in the conduction of the ReMonit study and had communicated with some of the participants related to this. All the authors have prior research experience in the field of digital health services. These prior experiences likely influenced our comprehension of the participants’ experiences, and some of the themes that surfaced during the interviews were recognizable.

### Implications for Practice

The significant technological advancements in health care in recent years have sparked a wave of optimism regarding remote patient monitoring. While previous studies have emphasized its positive aspects, our findings underscore various barriers that must be addressed, including issues related to acceptability, reliability, accessibility, and patients’ sense of security. Moreover, remote monitoring introduces new avenues for patient participation, collaboration, and self-efficacy, fundamentally reshaping health care delivery and traditional relationships. Adapting to these novel modes of interaction may pose challenges for patients, necessitating careful implementation of new follow-up strategies. Based on our results, we suggest the following implications for practice.

- Follow-up should be based on shared decision-making regarding the use of remote monitoring or patient-initiated care, with regular evaluations to assess users’ needs and preferences.
- Health care personnel should focus on increasing patients’ health literacy.
- The roles and responsibilities related to follow-up should be clarified and based on shared decision-making.
- Technology should feature a user-centered design that integrates individual patient preferences and requirements. Addressing patients’ concerns in future technological developments is crucial, aiming for user-friendly interfaces.

There are currently other recent studies as well as studies underway investigating similar interventions, which can further shed light on our research question [39,40].

### Implications for Future Research

Based on our results, we suggest the following implications for future research.

- Future research should emphasize diverse populations, considering variations in health literacy and digital competence as well as recruitment from both urban and remote areas.
- Future research should incorporate longitudinal studies to capture both the harms and benefits of remote monitoring over an extended period to capture experiences of remote care in phases with high disease activity.

### Conclusions

Remote monitoring and patient-initiated care can sufficiently meet the needs of patients with axSpA with low disease activity. When the disease has minimal impact on daily life, regular in-person consultations may be less necessary. These follow-up strategies can save patients time by reducing unnecessary visits and minimizing time off work. Additionally, using technology to manage well-controlled axSpA can enhance autonomy, self-management, and patient engagement.

However, patients emphasize that these approaches should not be one-size-fits-all. As disease activity, personal circumstances, and preferences evolve, care must be adapted accordingly. Human interaction, empathy, and support from health care professionals remain essential. Moreover, practical challenges and technological limitations can undermine the sense of security, making it crucial to address patients’ concerns in future technological developments, aiming for user-friendly and reliable interfaces.

## Appendix

Multimedia Appendix 1 COREQ Checklist.

Multimedia Appendix 2 Interview guide.

## Abbreviations

axSpA: axial spondyloarthritis
COREQ: Consolidated Criteria for Reporting Qualitative Research
CRP: C-reactive protein
PROM: patient-reported outcome measure
RCT: randomized controlled trial

## Data Availability

The datasets generated or analyzed during this study are not publicly available due to privacy concerns, to protect participants’ anonymity and ensure that sensitive information is not disclosed.
