# Supplementary material for: Perspectives on and Experiences With Remote Monitoring and Patient-Initiated Care Among Norwegian Patients With Axial Spondyloarthritis: Qualitative Study
Source: J Med Internet Res. 2025 Mar 28;27:e63569. doi: 10.2196/63569 (PMC11992489; doi:10.2196/63569)
Supplement: Multimedia Appendix 1 [file jmir_v27i1e63569_app1.pdf]

Multimedia Appendix 1: COREQ (COnsolidated criteria for REporting Qualitative research) Checklist

Title: Balancing distance and proximity: A qualitative study of Norwegian patients with Axial Spondyloarthritis' perspectives on and experiences with remote monitoring and patient-initiated care

Topic	Item no.	Guide questions/description	Reported on page no.
**Domain 1: Research team and reflexivity**			
Personal Characteristics			
Inter viewer/facilitator	1	Which author/s conducted the inter view or focus group?	5
Credentials	2	What were the researcher's credentials? E.g. PhD, MD	1
Occupation	3	What was their occupation at the time of the study?	5
Gender	4	Was the researcher male or female?	1
Experience and training	5	What experience or training did the researcher have?	5 and 13
Relationship with participants			
Relationship established	6	Was a relationship established prior to study commencement?	13
Participant knowledge of the interviewer	7	What did the participants know about the researcher? e.g. personal goals, reasons for doing the research	13
Interviewer characteristics	8	What characteristics were reported about the inter viewer/facilitator? e.g. Bias, assumptions, reasons and interests in the research topic	13
**Domain 2: study design**			
Theoretical framework			
Methodological orientation and Theory	9	What methodological orientation was stated to underpin the study? e.g. grounded theory, discourse analysis, ethnography, phenomenology, content analysis	5
Participant selection			
Sampling	10	How were participants selected? e.g. purposive, convenience, consecutive, snowball	4
Method of approach	11	How were participants approached? e.g. face-to-face, telephone, mail, email	4
Sample size	12	How many participants were in the study?	6
Non-participation	13	How many people refused to participate or dropped out? Reasons?	6
Setting			
Setting of data collection	14	Where was the data collected? e.g. home, clinic, workplace	5
Presence of non-participants	15	Was anyone else present besides the participants and researchers?	5
Description of sample	16	What are the important characteristics of the sample? e.g. demographic data, date	6
Data collection			
Interview guide	17	Were questions, prompts, guides provided by the authors? Was it pilot tested?	5
Repeat interviews	18	Were repeat inter views carried out? If yes, how many?	no
Audio/visual recording	19	Did the research use audio or visual recording to collect the data?	5
Field notes	20	Were field notes made during and/or after the inter view or focus group?	No
Duration	21	What was the duration of the inter views or focus group?	5
Data saturation	22	Was data saturation discussed?	5
Transcripts returned	23	Were transcripts returned to participants for comment and/or correction?	No
**Domain 3: analysis and findings**			

Data analysis			
Number of data coders	24	How many data coders coded the data?	5
Description of the coding tree	25	Did authors provide a description of the coding tree?	No
Derivation of themes	26	Were themes identified in advance or derived from the data?	5
Software	27	What software, if applicable, was used to manage the data?	6
Participant checking	28	Did participants provide feedback on the findings?	No
Reporting			
Quotations presented	29	Were participant quotations presented to illustrate the themes/findings? Was each quotation identified? e.g. participant number	6-10
Data and findings consistent	30	Was there consistency between the data presented and the findings?	Yes, 6-10
Clarity of major themes	31	Were major themes clearly presented in the findings?	Yes, 6-10
Clarity of minor themes	32	Is there a description of diverse cases or discussion of minor themes?	Yes, 6-10

Developed from: Tong A, Sainsbury P, Craig J. Consolidated criteria for reporting qualitative research (COREQ): a 32-item checklist for interviews and focus groups. International Journal for Quality in Health Care. 2007. Volume 19, Number 6: pp. 349 – 357

Once you have completed this checklist, please save a copy and upload it as part of your submission. DO NOT include this checklist as part of the main manuscript document. It must be uploaded as a separate file.
