# Supplementary material for: Perspectives on and Experiences With Remote Monitoring and Patient-Initiated Care Among Norwegian Patients With Axial Spondyloarthritis: Qualitative Study
Source: J Med Internet Res. 2025 Mar 28;27:e63569. doi: 10.2196/63569 (PMC11992489; doi:10.2196/63569)
Supplement: Multimedia Appendix 2 [file jmir_v27i1e63569_app2.docx]

# Multimedia Appendix 2: Interview Guide

##

## Patients with remote monitoring

### General questions

1. Can you tell us a little about yourself, your illness, and how you are managing it now?
2. You are a participant in this study; can you describe how you have been followed up while participating in the study?
   - How has it been?
   - Was it in line with your needs? Can you say a bit about why/why not?
3. Can you describe how the remote monitoring affected your daily life?
4. How would you say this type of follow-up affected your relationship with your healthcare providers?
5. Do you think differently about your illness now than you did before participating in the study? If so, please describe.
6. What experience do you have with using (similar?) technology from before?
7. Broadly speaking, what do you think about the use of this type of technology in healthcare?

### The functionality of the App

1. Can you tell us a bit about how you have used the App?
2. Have you contacted your healthcare provider outside of the regular reporting times? If yes, can you describe what happened?
3. Has your healthcare provider contacted you? If so, can you describe what happened?
4. How would you describe the questions you had to answer in the app?
   - Were any of the questions redundant?
   - Are there questions you think should have been included but weren't? Please describe.
5. What do you think about the frequency of the regular requests to answer questions?
   - What do you think would be an ideal follow-up frequency?
   - What do you think about the time of day the notifications come?
6. What do you think about the user-friendliness/functionality of the app?
   - Are there elements you think work well or less well?
7. What do you think is important for this type of tool to work well?
8. Would you like to continue with remote monitoring after the study ends? Why/why not?

### Concluding questions

1. Of everything we've talked about today, what do you think is important to highlight?
2. Is there anything else you feel strongly about that you would like to discuss?

## Patients with patient-initiated care

### General questions

1. Can you tell us a little about yourself, your illness, and how you are managing it now?
2. You are a participant in this study; can you describe how you have been followed up while participating in the study?
   - How has it been?
   - Was it in line with your needs? Can you say a bit about why/why not?
3. Can you describe how having patient-initiated care has affected your daily life?
4. How would you say this type of follow-up affected your relationship with your healthcare providers?
5. Do you think differently about your illness now than you did before participating in the study? If so, please describe.
6. Have you contacted your healthcare provider outside of the regular appointments? If yes, can you describe what happened?
7. Would you like to continue with patient-initiated care after the study ends? Why/why not?

### The functionality of the App

1. You downloaded the MyDignio app; can you describe how you used the app?
2. How would you describe the questions you had to answer in the app?
   - - Were any of the questions redundant?
     - Are there questions you think should have been included but weren't? Please describe.
3. What do you think about the frequency of the regular requests to answer questions?
   - - What do you think would be an ideal follow-up frequency?
     - What do you think about the time of day do the app notifications came?
4. What do you think about the user-friendliness/functionality of the app?
   - - Are there elements you think work well or less well?
5. What do you think is important for this type of tool to work well?
6. Would you like to continue with the app after the study ends? Why/why not?
7. What experience do you have with using (similar?) technology from before?
8. Broadly speaking, what do you think about the use of this type of technology in healthcare?

### Concluding questions

1. Of everything we've talked about today, what do you think is important to highlight?
2. Is there anything else you feel strongly about that you would like to discuss?
